# Calomel and Tartar Emetic as Remedial Agents

**Published:** 1863-08

**Authors:** 


### EDITORIAL DEPARTMENT.

### CALOMEL AND TARTAR EMETIC AS REMEDIAL AGENTS.

Surgeon General's Office,
Washington City, June 12, 1862.

Dear Sir:—Desiring to obtain the opinions of the more eminent members of the Medical profession to the indiscriminate use of Calomel and Tartarized Antimony, I have the honor to request that you will answer the following questions:

1st—To what extent do you prescribe Calomel and Tartar Emetic in your practice?

2d—Do you regard these agents as indispensable in the treatment of disease?

3d—In view of the facts that a large number of the Medical Officers of the Army are young and inexperienced, and that soldiers cannot in the field be placed beyond the influence of atmospheric vicissitues and exposure whilst undergoing medical treatment, would you recommend that the medicines in question be issued to Army Medical Officers, except, as at present, upon special requisition?

4th—Do you or do you not think that more harm than good has resulted from the use of Calomel and Tartar Emetic as medicines?

It should be stated that the following mercurials are at present on the Supply Table, viz;

Hydrargyri chloridum corrosivum; Hydrargyri iodidum flavum; Hydrargyri oxidum rubrum; Hydrargyri pilulæ; Hydrargyri unguentum; Hydrargyri nitratis unguentum; Pilulæ catharticæ compositæ; and that it is provided by paragraph 13, of Circular No. 7, dated Surgeon-General's Office, May 7, 1863, which contains the Supply Table, and which refers to the manner of obtaining medical supplies, that "it is not the design of the Department to confine Medical Officers absolutely to that table, either in variety or quantity, but only to establish a standard for their guidance in making requisitions for supplies, leaving individual preferences to be indulged at the discretion of the Medical-Director or the Surgeon-General.—Neither is it supposed that the quantities of the table will always meet the necessities of unusual emergencies, as during epidemics, or in unhealthy seasons and localities; and Medical Officers who allow their supplies to be exhausted through any such contingencies, without timely notice of their approaching necessities, will be held to a strict accountability."

I am, sir, very respectfully,
Your obedient servant,
William A. Hammond,
*Surgeon-General U. S. A.*

Upon the order of the Surgon-General striking Calomel and Tartar Emetic from the Medical Supply Table of the Army, or upon the subjects of inquiry above presented to the distinguished physicians of the Army Medical Staff, we have never as yet ventured an expression. It has seemed to us that every possible view had already been presented, and that unless we could add some important item, our readers would excuse us in keeping unbroken silence. We have felt willing that this matter should rest upon its own merits, and have not been distressed that some Homœopathic, Eclectic, Thompsonian, Spiritual pretender, should become jubilant over the order, and announce to bar-room congregations that "Calomel Doctors had been totally squelched by order of the Surgeon-General." It has never once occurred to us that the soldiers would suffer greatly from the privation, or that the physicians of the army would be obliged to resign on account of the indignity heaped upon them by this order. Surgery is so much done by "order" that army physicians have become accustomed to "orders," and do not take it so hard as those in civil practice, who take advice, but would be entirely unable to take "orders." It indicates that potent medicines have been abused by the inexperienced and incapable medical men in the army, and that this abuse, and misuse, was so great that it could better be dispensed with altogether. We cannot say much in favor of the taste exhibited in the manner of abating these dangerous remedies, and certainly had it not come to be the general style of doing things, we could hardly overlook and excuse so summary and impolite treatment. If the Surgeon-General was a physician only, we would abuse him for not being more gentlemanly, but since he is an Officer in the Army, we must let him do his duty in his own way.

If calomel was used, to any great extent in the treatment of the diseases of the soldiers; if, as he alleges, "its injurious effects were observable in the hospitals of the army," and ptyalism was as common among the inmates, as we have reason to fear, then we are glad that the order was issued; glad the Surgeon-General had enough decision of character and manliness of purpose to stop it short off, and heartily wish other officers in the army would correct abuses in their departments as summarily. Of tartar emetic we have nothing to say, only that we think it well for the soldiers that they are not to have any more of it, and have only been sorry that the order excluding it from the army, could not be extended, excluding it from almost universal abuse. This is not because it is always injurious, but because as used it does vastly more harm than good, and there has never yet been found arguments or evidences sufficiently powerful or extensively diffused, to prevent its continual misuse.

This suggestion which has somewhere been made of excluding these articles by a careful revision of the medical supply table, pleases us very much. It would have been a polite way of doing the thing and afforded opportunity of excluding at least one-half of the remaining articles; however if they are left *on the table*, they will do no harm.

The replies which will be given to the inquiries above will be interesting and important, since it is expected that the leading Army Surgeons and Medical Inspectors will respond to this circular. Civil physicians are not in situation to discuss these questions with a view to their proper solution, and it is not supposed that they will be invited to give their opinions.

The universal employment of these two articles by civil practitioners is no doubt one reason of abuse in the army, since most of our military surgeons were recently in civil practice, and to great extent would adopt similar treatment in the army. As to the propriety of using these or similar articles in the treatment of disease, there can be no doubt; the profession will be found unanimous in the opinion that they are indispensable to the successful practice of medicine. It appears that this is conceded by the Surgeon-General, and that it is on the ground of abuse, and not of proper use, that the order of exclusion is based. The American Medical Association passed severe resolutions upon this subject, and yet we mistrust that time, and testimony, will tell tales which will go very far towards justifying this order. The fourth inquiry which is made, is a little remarkable. If our most experienced and observing physicians were asked: Do you or do you not think that more harm than good has resulted from the use of medicine? what would be the reply? and what would be the import if they should express the opinion that medicine had done, and was doing, more harm than good? It would certainly be no argument against judicious and rational medication, and would simply declare that the misuse is so great as to outweigh in the aggregate all good which is accomplished. It would reflect nothing upon legitimate medication, or weaken in the least our confidence in its beneficial effects. All other powerful and popular articles of the materia medica, capable of abuse, would as appropriately come under inquiry. What reply *should* the Surgeon-General receive if he wishes to know the same of stimulants? the very article which he estimates so highly. We wish he would ask this question as earnestly and would extend the inquiry to eminent, practical men in civil practice.—If the reply should go back to him, that it has killed more officers and men in the army than both disease and battle, would he issue an order for its total exclusion from the supply table and the lines of the army? if he would, he may as well issue his order. If it has not done this in the army, it has done it in civil life, and has disabled more men and rendered them unfit for military duty, or any other duty, than have been in both armies, on service, killed, wounded and missing. Opium, the most valuable medicine in the whole list of the materia medica, is also liable to great abuse, and if we estimate its uses and abuses, it is quite possible that the injuries of the one would quite outdo the benefits of the other. We have not time or space to devote to a discussion of this subject, and have referred to it mainly because it is so unfashionable to let it alone.

